# *KCNA5 *gene is not confirmed as a systemic sclerosis-related pulmonary arterial hypertension genetic susceptibility factor

**DOI:** 10.1186/ar4124

**Published:** 2012-12-27

**Authors:** Lara Bossini-Castillo, Carmen P Simeon, Lorenzo Beretta, Jasper Broen, Madelon C Vonk, José Luis Callejas, Patricia Carreira, Luis Rodríguez-Rodríguez, Rosa García-Portales, Miguel A González-Gay, Ivan Castellví, María Teresa Camps, Carlos Tolosa, Esther Vicente-Rabaneda, María Victoria Egurbide, Annemie J Schuerwegh, Roger Hesselstrand, Claudio Lunardi, Jacob M van Laar, Paul Shiels, Ariane Herrick, Jane Worthington, Christopher Denton, Timothy RDJ Radstake, Carmen Fonseca, Javier Martin

**Affiliations:** 1 Instituto de Parasitología y Biomedicina López-Neyra, IPBLN-CSIC, Avenida del Conocimiento s/n, Granada, 18100, Spain; 2 Servicio de Medicina Interna, Hospital Valle de Hebron, Passeig de la Vall d'Hebron, 119-129, Barcelona, 08035, Spain; 3 Referral Center for Systemic Autoimmune Diseases Fondazione IRCCS Ca' Granda Ospedale Maggiore Policlinico di Milano, via Francesco Sforza 28, Milan, 20122, Italy; 4 Department of Rheumatology & Clinical Immunology, University Medical Center, Heidelberglaan 100, 3485 CX Utrecht, The Netherlands; 5 Laboratory of Translational Immunology, University Medical Center, Heidelberglaan 100, 3485 CX Utrecht, The Netherlands; 6 Department of Rheumatology, Radboud University Nijmegen Medical Centre, Geert Grooteplein-Zuid 10, Nijmegen, 6525 GA, The Netherlands; 7 Servicio de Medicina Interna, Hospital Clínico Universitario, Avenida Doctor Olóriz 16, Granada, 18012, Spain; 8 Servicio de Reumatología, Hospital Universitario 12 de Octubre, Avenida de Córdoba, s/n, Madrid, 28041, Spain; 9 Servicio de Reumatología, Hospital Clínico San Carlos, C/Profesor Martín Lagos s/n, Madrid, 28040, Spain; 10 Servicio de Reumatología, Hospital Universitario Virgen de la Victoria, Campus Universitario Teatinos s/n, Málaga, 29010, Spain; 11 Servicio de Reumatología, Hospital Universitario Marqués de Valdecilla, IFIMAV, Avenida Valdecilla 25, Santander, 39008, Spain; 12 Servicio de Reumatología, Hospital de la Santa Creu i Sant Pau, C/Sant Antoni Maria Claret 167, Barcelona, 08025, Spain; 13 Servicio de Medicina Interna, Hospital Carlos Haya, Avenida Carlos Haya s/n, Málaga, 29010, Spain; 14 Servicio de Medicina Interna, Hospital Parc Tauli, Parc del Taulí 1, Sabadell, 08208, Spain; 15 Servicio de Reumatología, Hospital Universitario de la Princesa, C Diego de León 62, Madrid, 28006, Spain; 16 Servicio de Medicina Interna, Hospital de Cruces, Plaza de Cruces 2, Barakaldo, 48903, Spain; 17 Department of Rheumatology, Leiden University Medical Center, Albinusdreef 2, Leiden, 2333 ZA, The Netherlands; 18 Department of Rheumatology, Lund University, Paradisgatan 2, Lund, SE-221 00, Sweden; 19 Department of Medicine, Università degli Studi di Verona, Via dell'Artigliere 19, Verona, 37129, Italy; 20 Institute of Cellular Medicine, Newcastle University, Framlington Place, Newcastle upon Tyne, Newcastle, NE2 4HH, UK; 21 Centre for Rheumatic Diseases, Glasgow Royal Infirmary, 84 Castle Street, Glasgow G4 0SF, UK; 22 Arthritis Research UK Epidemiology Unit, The University of Manchester, Manchester Academic Health Science Centre, Stopford Building, Oxford Road, Manchester, M13 9PT, UK; 23 Centre for Rheumatology, Royal Free and University College Medical School, University College London, Royal Free Campus, Rowland Hill Street, London, NW3 PF, UK

## Abstract

**Introduction:**

Potassium voltage-gated channel shaker-related subfamily member 5 (KCNA5) is implicated in vascular tone regulation, and its inhibition during hypoxia produces pulmonary vasoconstriction. Recently, a protective association of the *KCNA5 locus *with systemic sclerosis (SSc) patients with pulmonary arterial hypertension (PAH) was reported. Hence, the aim of this study was to replicate these findings in an independent multicenter Caucasian SSc cohort.

**Methods:**

The 2,343 SSc cases (179 PAH positive, confirmed by right-heart catheterization) and 2,690 matched healthy controls from five European countries were included in this study. Rs10744676 single-nucleotide polymorphism (SNP) was genotyped by using a TaqMan SNP genotyping assay.

**Results:**

Individual population analyses of the selected *KCNA5 *genetic variant did not show significant association with SSc or any of the defined subsets (for example, limited cutaneous SSc, diffuse cutaneous SSc, anti-centromere autoantibody positive and anti-topoisomerase autoantibody positive). Furthermore, pooled analyses revealed no significant evidence of association with the disease or any of the subsets, not even the PAH-positive group. The comparison of PAH-positive patients with PAH-negative patients showed no significant differences among patients.

**Conclusions:**

Our data do not support an important role of *KCNA5 *as an SSc-susceptibility factor or as a PAH-development genetic marker for SSc patients.

## Introduction

Systemic sclerosis (SSc) is a life-threatening fibrotic connective tissue disorder that affects the skin and different internal organs [1]. The 10-year survival of SSc patients reaches only 63%, with pulmonary involvement the leading cause of death [2]. SSc is a complex disorder in which the environmental triggers and genetic susceptibility factors co-act in the development and maintenance of the disease [3]. As a clearer picture of the genetic component of this disease is being revealed, interest in genetic markers for specific clinical features, especially lung involvement, is increasing. An SSc phenotype-restricted genome-wide analysis was carried out recently [4]. Remarkably, two of the four new SSc genetic-susceptibility markers identified in the previously mentioned study might play a relevant role in the SSc-related fibrotic process, *SOX5 *and *NOTCH4 *[4]. However, no such strategy has yet been considered for pulmonary involvement, and only some studies have reported significant genetic association with SSc-related lung involvement [5-9]. Furthermore, only the association of *CD226 *with pulmonary fibrosis has been independently replicated [10]. Wipff *et al. *[11] recently described the association of potassium voltage-gated channel shaker-related subfamily member 5 (*KCNA5*) with SSc-related pulmonary arterial hypertension (PAH).

Potassium voltage-gated channels (Kv channels) are homo- or heterotetramers of structural α-subunits and regulatory β-subunits, which control potassium (K) flux. K balance is known to regulate the apoptotic cell shrinkage, a main event in the apoptotic process [12]. Specifically, KCNA5 participates in pulmonary artery smooth muscle cell (PASMC) apoptosis control [13]. It has been reported that overexpression of the *KCNA5 *gene induces accelerated K efflux and increases caspase-3 proteolytic activity, promoting apoptosis [13].

KCNA5 is also involved in membrane-potential maintenance and vascular-tone regulation [14]. Hypoxic conditions produce a specific inhibition of KCNA5 in the PASMCs, causing pulmonary vasoconstriction [15-17]. Moreover, it has been reported that primary pulmonary hypertension patients have an intrinsic reduced level of *KCNA5 *mRNA in their PASMCs, and this characteristic might play an important role in the pathogenesis of the disease [18].

The role of *KCNA5 *genetic polymorphisms in pulmonary disease also was explored. Remillard *et al. *[19] analyzed the influence of *KCNA5 *genetic variants in IPAH, and showed that different single-nucleotide polymorphisms (SNPs) were related to abnormal function or drug responsiveness. Recently, the importance of *KCNA5 *variants in SSc-related PAH was analyzed [11]. In the previously mentioned study, the association of *KCNA5 *with SSc and specifically with PAH-positive (PAH^+^) patients was described [11]. Moreover, the rs10744676 polymorphism was reported as the variant underlying the observed association [11].

In light of the previous evidence, the main goal of this report is to replicate the association of *KCNA5 *rs10744676 polymorphism with SSc and SSc-related PAH in an independent European population of Caucasian ancestry.

## Materials and methods

### Subjects

Our study comprised 2,343 SSc cases and 2,690 controls from Spain, The Netherlands, Italy, Sweden, and The United Kingdom. All the individuals included in this report were of Caucasian ancestry. Patients were classified as having limited (lcSSc) or diffuse SSc (dcSSc), according to their skin involvement, as defined by LeRoy *et al. *[20]. The serologic subgroup stratification of the patients was based on the presence of SSc-associated autoantibodies (anti-centromere antibodies (ACAs) and anti-topoisomerase (ATA)). Pulmonary fibrosis was diagnosed by the presence of interstitial abnormalities in high-resolution computed tomography (HRCT). Patients defined as PAH^+ ^showed a mean resting pulmonary artery pressure > 25 mm Hg and a pulmonary capillary wedge pressure ≤15 mm Hg, at the time of a right-heart catheterization [11]. The control population consisted of unrelated healthy individuals recruited in the same geographic regions as the SSc patients and matched by age, sex, and ethnicity with the SSc-patient groups. Approval of local ethical committees was obtained from all the participating centers (Comité de Bioética del Consejo Superior de Investigaciones Científicas, Comitato Etico Azienda Ospedaliera Universitaria Integrata di Verona, Local Ethics Committee of the Radboud University Nijmegen Medical Centre, Medische Ethische Commissie Leids Universitair Medisch Centrum, The regional Ethical Review Board in Lund, Local Research Ethics Committee at Glasgow Royal Infirmary, U.O. Comitato di Etica e Sperimentazione Farmaci Fondazione IRCCS Ca' Granda-Ospedale Maggiore Policlinico di Milano, Local Research Ethics Committee at Glasgow Royal Infirmary, Royal Free Hospital and Medical School Research Ethics Committee, Manchester University Research Ethics Committee). Written informed consent was required for both patients and controls to be included in the study.

### Genotyping and statistical analysis

The rs10744676 *KCNA5 *biallelic variant was analyzed with TaqMan SNP genotyping assay in a 7900HT Real-Time polymerase chain reaction (PCR) system from Applied Biosystems by following the manufacturer's suggestions (Foster City, CA, USA).

None of the included cohorts showed significant deviation from Hardy-Weinberg equilibrium (HWE) (*P *value significance threshold, 0.05). Significance for the allelic model in the individual cohort analyses was calculated by 2 × 2 contingency tables and the Fisher Exact test or χ^2 ^when necessary. Odds ratios (ORs) were calculated according to the Woolf method. A Breslow-Day test (BD test) was performed to assess the homogeneity of the association among populations, and pooled analysis under a Cochran-Mantel-Haenszel fixed-effect model was used to analyze jointly all the included cohorts. Statistical analyses were performed as implemented in PLINK (v1. 07) software package [21]. Power was calculated by using the software Power Calculator for Genetic Studies 2006 and assuming an additive model and previously reported minor allele frequency (MAF) and ORs [22].

## Results

The power of our replication study in each stratified analysis is summarized in Table 1. We emphasize that the size of our pooled PAH^+ ^patient subgroup represents the largest SSc-related PAH^+ ^cohort analyzed to date (*n *= 179). The power of the study of this clinical feature in our population to detect an association equivalent to that previously reported by Wipff *et al. *is 95%, at the 5% significance level.

**Table 1:** Overall statistical power of the study for *KCNA5 *rs10744676 SNP in each analyzed disease subtype at the 5% significance level assuming an additive effect model and a minor allele (rs10744676*C) frequency = 0.10 (MAF_CEU_)

	Phenotype						
	
Statistical power (%)	SSc	lcSSc	dcSSc	ACA^+^	ATA^+^	Fib^+^	PAH^+^
OR = 0.62	100	100	99	100	96	99	95^a^
OR = 0.48	100	100	100	100	100	100	100^b^
OR = 0.79	96	91	67	77	59	71	45^c^

^a^Reference OR, 0.62, except for the PAH^+ ^group, in which OR = 0.47. ^b^Reference OR (lower previously reported 95% CI) = 0.48, except for the PAH^+ ^group, in which OR = 0.32. ^c^Reference OR (upper previously reported 95% CI) = 0.79, except for the PAH^+ ^group, in which OR = 0.71. SSc, systemic sclerosis; lcSSc, limited cutaneous systemic sclerosis; dcSSc, diffuse cutaneous systemic sclerosis; ACA^+^, anti-centromere autoantibody-positive patients; ATA^+^, anti-topoisomerase autoantibody-positive patients; Fib^+^, lung fibrosis-positive patients (HRCT); PAH^+^, pulmonary arterial hypertension-positive patients (right-heart catheterization).

Table 2 shows the results of the comparison of the complete set of SSc and each of the previously defined subgroups with the healthy control group. As observed in the table, the analyzed polymorphism showed no significant association with either the disease or any of the subsets, not even the PAH-positive group. Furthermore, no significant association was observed in the individual population analyses, either in the whole disease comparison or in the different subphenotypes (see Additional file 1, Table S1). The different cohorts showed a high interpopulation combinability, as can be observed in the Breslow-Day test results (Table 2).

**Table 2:** Pooled analysis and stratified analyses of SSc patients and healthy controls for rs10744676 genetic variant, located in the *KCNA5 *gene

	Genotype, *n *(%)				Allele test		
			
Subgroup (*n*)	C/C	C/T	T/T	MAF (%)	*P*_MH_	OR (95% CI)	*P*_BD_
Controls (*n *= 2,690)	37 (1.38)	597 (22.19)	2,056 (76.43)	12.47			
SSc (*n *= 2,343)	43 (1.84)	471 (20.10)	1,829 (78.06)	11.89	0.49	0.96 (0.85-1.08)	0.53
lcSSc (*n *= 1,642)	31 (1.89)	327 (19.91)	1,284 (78.20)	11.85	0.48	0.95 (0.83-1.09)	0.63
dcSSc (*n *= 701)	12 (1.71)	144 (20.54)	545 (77.75)	11.98	0.80	0.98 (0.81-1.17)	0.59
ACA^+ ^(*n *= 931)	18 (1.93)	197 (21.16)	716 (76.91)	12.51	0.49	1.06 (0.90-1.24)	0.96
ATA+ (*n *= 568)	8 (1.41)	117 (20.60)	443 (77.99)	11.71	0.85	0.98 (0.80-1.20)	0.94
Fib^+ ^(*n *= 771)	14 (1.82)	154 (19.97)	603 (78.21)	11.80	0.34	0.92 (0.77-1.09)	0.92
PAH^+ ^(*n *= 179)	2 (1.12)	37 (20.67)	140 (78.21)	11.45	0.44	0.88 (0.63-1.23)	0.70

MAF, minor allele frequency; *P*_MH_, Mantel-Haenszel test under fixed-effect *P *values. Controls are used as reference for all comparisons, and *P *values have been calculated for the allelic model. OR, odds ratio for the minor allele; 95% CI, 95% confidence interval; SSc, systemic sclerosis; lcSSc, limited cutaneous systemic sclerosis; dcSSc, diffuse cutaneous systemic sclerosis; ACA^+^, anti-centromere autoantibody-positive patient; ATA^+^, anti-topoisomerase autoantibody-positive patient; Fib^+^, lung fibrosis-positive patient (HRCT); PAH^+^, pulmonary arterial hypertension-positive patients (right-heart catheterization).

The minor allele in the Spanish and Italian control populations included in this study was less frequent than that in the pooled population described by the Wipff *et al. *(rs10744676*C Frequency_Spain _= 10.48%; rs10744676*C Frequency_Italy _= 10.00%; rs10744676*C Frequency_pooled_Wipff _= 14.7%). However, these frequencies are in concordance with those reported for the HapMap CEU population (MAF_HapMap_CEU _= 10.00%). Moreover, MAF differences have been reported in different European populations (MAF_1000Genomes_CEU _= 17.64%, MAF_1000Genomes_GBR _= 15.73%, MAF_1000Genomes_FIN _= 13.98%, MAF_1000Genomes_TSI _= 8.16%).

In addition, the analysis of PAH^+ ^versus PAH^- ^patients revealed no phenotype-restricted association in this subgroup (*P*_MH _= 0.59; OR, 0.91; 95% CI, 0.64 to 1.28).

## Discussion

Despite the previous findings of an association of the rs10744676 *KCNA5 *genetic variant with PAH^+ ^SSc patients, our data do not corroborate the reported protective effect of the minor allele of this polymorphism on SSc-related PAH.

It is worth mentioning that the prevalence of right-heart catheterization-confirmed PAH in our pooled population (7.64%) is consistent with previous reports [11]. However, SSc is a chronic progressive disease, and some patients classified as PAH^- ^might develop PAH in the future. Therefore, we consider this issue a limitation of our study and similar reports. In this context, we point out that the mean disease duration for the patient group included in this study (mean disease duration, 13.9 ± 14.4 years, as reported in Bossini-Castillo *et al. *[23]) was higher than that in Wipff *et al. *[11] (11.9 ± 9.4 years in the discovery cohort and 13.9 ± 14.4 years in the replication), which might influence the observed differences.

The PAH^+ ^cohort studied in this report reached a 95% estimated statistical power to detect an association equivalent to that observed by Wipff *et al. *within this subgroup (OR, 0.47, in the comparison between PAH^+ ^SSc patients and controls of the discovery phase and two replication steps [11]). Hence, we consider that the lack of replication observed in our study is unlikely to be caused by a type II error (false negative) because of a reduced statistical power. However, autoimmune-associated variants usually show modest degrees of risk, especially in the case of non-HLA loci [24]. Wipff *et al. *reported an OR for *KCNA5 *rs10744676 polymorphism that was remarkably more protective than the SSc genetic-association standards for non-HLA loci (that is, modest ORs between 0.70 and 1). Thus, we reflect that if the influence of rs10744676 in the SSc-patient genetic predisposition to PAH is modest, the statistical power to detect a possible association in the PAH^+ ^cohort analyzed in the present article might be insufficient, and a possible modest effect of *KCNA5 *rs10744676 might be overlooked (Table 1).

In addition, it is established that PAH is more frequent in ACA^+ ^patients [1], and despite the suggestive association with PAH^+ ^patients, no significant association with the ACA^+ ^subphenotype was identified in the previous SSc study [11] or in this report. This fact is also consistent with a lack of association of the selected polymorphism with PAH development.

Although rs10744676 might have a functional role in *KCNA5 *expression because of its location in its putative promoter, no evidence confirms a functional role for this variant. Therefore, we speculate that the previously mentioned association with SSc could be the consequence of a tagged causal variant yet to be discovered. Moreover, because PAH onset time has not been considered for the analyses, it might act as a confounding factor in the discrepant results.

## Conclusions

In summary, our data do not support an important role of rs10744676 as a PAH genetic marker in SSc patients.

## Abbreviations

ACA: anti-centromere autoantibodies; ATA: anti-topoisomerase antibodies; BD test: Breslow-Day test; *CD226*: cluster of differentiation 226; CEU: Utah residents with ancestry from northern and western Europe; dcSSc: diffuse cutaneous systemic sclerosis; hRCT: High-resolution computed tomography; HWE: Hardy-Weinberg equilibrium; K: potassium; *KCNA5*: potassium voltage-gated channel shaker-related subfamily member 5; Kv channels: potassium voltage-gated channels; lcSSc: limited cutaneous systemic sclerosis; MAF: minor allele frequency; *NOTCH4*: Notch (*Drosophila*) homologue 4; OR: odds ratio; PAH: pulmonary arterial hypertension; PASMC: pulmonary artery smooth muscle cell; PCR: polymerase chain reaction; SNP: single-nucleotide polymorphism; *SOX5*: SRY (sex-determining region Y)-box 5; SSc: systemic sclerosis.

## Competing interests

The authors declare that they have no competing interests.

## Authors' contributions

LBC contributed to the analysis and interpretation of data and to the drafting of the manuscript. CPS, LB, and JB participated in the acquisition of data and the drafting of the manuscript. CF, TRDJR, and JM contributed to the conception and design of the study and critically revised the manuscript. MCV, JLC, PC, LRR, RGP, MAGG, IC, MTC, CT, EVR, MVE, AJS, RH, CL, JMvL, PS, AH, JW, CD, and the Spanish Scleroderma Group were involved in the acquisition of data and the revision of the manuscript. All authors read and approved the final manuscript.

## Supplementary Material

Additional file 1 **Genotype and minor allele frequencies of *KCNA5 *rs10744676 genetic variant in five European cohorts**. This file contains Table S1, showing the genotype and allele distributions of *KCNA5 *rs10744676 genetic variant in five European cohorts (2,343 SSc cases and 2,690 controls). Click here for file

## References

[B1] Gabrielli A Avvedimento EV Krieg T Scleroderma N Engl J Med 2009 14 1989 2003 10.1056/NEJMra0806188 19420368

[B2] Steen VD Medsger TA Changes in causes of death in systemic sclerosis, 1972-2002 Ann Rheum Dis 2007 14 940 944 10.1136/ard.2006.066068 17329309 PMC1955114

[B3] Martin JE Bossini-Castillo L Martin J Unraveling the genetic component of systemic sclerosis Hum Genet 2012 14 1023 1037 10.1007/s00439-011-1137-z 22218928

[B4] Gorlova O Martin JE Rueda B Koeleman BP Ying J Teruel M Diaz-Gallo LM Broen JC Vonk MC Simeon CP Alizadeh BZ Coenen MJ Voskuyl AE Schuerwegh AJ van Riel PL Vanthuyne M van 't Slot R Italiaander A Ophoff RA Hunzelmann N Fonollosa V Ortego-Centeno N Gonzalez-Gay MA Garcia-Hernandez FJ Gonzalez-Escribano MF Airo P van Laar J Worthington J Hesselstrand R Smith V Identification of novel genetic markers associated with clinical phenotypes of systemic sclerosis through a genome-wide association strategy PLoS Genet 2011 14 e1002178 10.1371/journal.pgen.1002178 21779181 PMC3136437

[B5] Dieude P Guedj M Wipff J Ruiz B Hachulla E Diot E Granel B Sibilia J Tiev K Mouthon L Cracowski JL Carpentier PH Amoura Z Fajardy I Avouac J Meyer O Kahan A Boileau C Allanore Y STAT4 is a genetic risk factor for systemic sclerosis having additive effects with IRF5 on disease susceptibility and related pulmonary fibrosis Arthritis Rheum 2009 14 2472 2479 10.1002/art.24688 19644887

[B6] Dieude P Guedj M Wipff J Ruiz B Riemekasten G Airo P Melchers I Hachulla E Cerinic MM Diot E Hunzelmann N Caramaschi P Sibilia J Tiev K Mouthon L Riccieri V Cracowski JL Carpentier PH Distler J Amoura Z Tarner I Avouac J Meyer O Kahan A Boileau C Allanore Y NLRP1 influences the systemic sclerosis phenotype: a new clue for the contribution of innate immunity in systemic sclerosis-related fibrosing alveolitis pathogenesis Ann Rheum Dis 2011 14 668 674 10.1136/ard.2010.131243 21149496

[B7] Dieude P Guedj M Wipff J Ruiz B Riemekasten G Matucci-Cerinic M Melchers I Hachulla E Airo P Diot E Hunzelmann N Cabane J Mouthon L Cracowski JL Riccieri V Distler J Meyer O Kahan A Boileau C Allanore Y Association of the TNFAIP3 rs5029939 variant with systemic sclerosis in the European Caucasian population Ann Rheum Dis 2010 14 1958 1964 10.1136/ard.2009.127928 20511617

[B8] Hoshino K Satoh T Kawaguchi Y Kuwana M Association of hepatocyte growth factor promoter polymorphism with severity of interstitial lung disease in Japanese patients with systemic sclerosis Arthritis Rheum 2011 14 2465 2472 10.1002/art.30415 21520010

[B9] Dieude P Guedj M Truchetet ME Wipff J Revillod L Riemekasten G Matucci-Cerinic M Melchers I Hachulla E Airo P Diot E Hunzelmann N Mouthon L Cabane J Cracowski JL Riccieri V Distler J Amoura Z Valentini G Camaraschi P Tarner I Frances C Carpentier P Brembilla NC Meyer O Kahan A Chizzolini C Boileau C Allanore Y Association of the CD226 Ser(307) variant with systemic sclerosis: evidence of a contribution of costimulation pathways in systemic sclerosis pathogenesis Arthritis Rheum 2011 14 1097 1105 10.1002/art.30204 21162102

[B10] Bossini-Castillo L Simeon CP Beretta L Broen JC Vonk MC Rios-Fernandez R Espinosa G Carreira P Camps MT Castillo MJ Gonzalez-Gay MA Beltran E Carmen Freire MD Narvaez J Tolosa C Witte T Kreuter A Schuerwegh AJ Hoffmann-Vold AM Hesselstrand R Lunardi C van Laar JM Chee MM Herrick A Koeleman BP Denton CP Fonseca C Radstake TR Martin J A multicenter study confirms CD226 gene association with systemic sclerosis-related pulmonary fibrosis Arthritis Res Ther 2012 14 R85 10.1186/ar3809 22531499 PMC3446459

[B11] Wipff J Dieude P Guedj M Ruiz B Riemekasten G Cracowski JL Matucci-Cerinic M Melchers I Humbert M Hachulla E Airo P Diot E Hunzelmann N Caramaschi P Sibilia J Valentini G Tiev K Girerd B Mouthon L Riccieri V Carpentier PH Distler J Amoura Z Tarner I Degano B Avouac J Meyer O Kahan A Boileau C Allanore Y Association of a KCNA5 gene polymorphism with systemic sclerosis-associated pulmonary arterial hypertension in the European Caucasian population Arthritis Rheum 2010 14 3093 3100 10.1002/art.27607 20556823

[B12] Bortner CD Hughes FM Jr Cidlowski JA A primary role for K^+ ^and Na^+ ^efflux in the activation of apoptosis J Biol Chem 1997 14 32436 32442 10.1074/jbc.272.51.32436 9405453

[B13] Brevnova EE Platoshyn O Zhang S Yuan JX Overexpression of human KCNA5 increases IK V and enhances apoptosis Am J Physiol Cell Physiol 2004 14 C715 722 10.1152/ajpcell.00050.2004 15140747

[B14] Yuan XJ Voltage-gated K^+ ^currents regulate resting membrane potential and [Ca^2+^]_i _in pulmonary arterial myocytes Circ Res 1995 14 370 378 10.1161/01.RES.77.2.370 7542182

[B15] Archer SL Souil E Dinh-Xuan AT Schremmer B Mercier JC El Yaagoubi A Nguyen-Huu L Reeve HL Hampl V Molecular identification of the role of voltage-gated K^+ ^channels, Kv1.5 and Kv2.1, in hypoxic pulmonary vasoconstriction and control of resting membrane potential in rat pulmonary artery myocytes J Clin Invest 1998 14 2319 2330 10.1172/JCI333 9616203 PMC508821

[B16] Platoshyn O Brevnova EE Burg ED Yu Y Remillard CV Yuan JX Acute hypoxia selectively inhibits KCNA5 channels in pulmonary artery smooth muscle cells Am J Physiol Cell Physiol 2006 14 C907 C916 16236819 10.1152/ajpcell.00028.2005 PMC1363730

[B17] Coppock EA Martens JR Tamkun MM Molecular basis of hypoxia-induced pulmonary vasoconstriction: role of voltage-gated K^+ ^channels Am J Physiol Lung Cell Mol Physiol 2001 14 L1 L12 11404238 10.1152/ajplung.2001.281.1.L1

[B18] Yuan XJ Wang J Juhaszova M Gaine SP Rubin LJ Attenuated K^+ ^channel gene transcription in primary pulmonary hypertension Lancet 1998 14 726 727 10.1016/S0140-6736(05)78495-6 9504523

[B19] Remillard CV Tigno DD Platoshyn O Burg ED Brevnova EE Conger D Nicholson A Rana BK Channick RN Rubin LJ O'Connor DT Yuan JX Function of Kv1.5 channels and genetic variations of KCNA5 in patients with idiopathic pulmonary arterial hypertension Am J Physiol Cell Physiol 2007 14 C1837 C1853 17267549 10.1152/ajpcell.00405.2006

[B20] LeRoy EC Black C Fleischmajer R Jablonska S Krieg T Medsger TA Jr Rowell N Wollheim F Scleroderma (systemic sclerosis): classification, subsets and pathogenesis J Rheumatol 1988 14 202 205 3361530

[B21] Purcell S Neale B Todd-Brown K Thomas L Ferreira MA Bender D Maller J Sklar P de Bakker PI Daly MJ Sham PC PLINK: a tool set for whole-genome association and population-based linkage analyses Am J Hum Genet 2007 14 559 575 10.1086/519795 17701901 PMC1950838

[B22] Skol AD Scott LJ Abecasis GR Boehnke M Joint analysis is more efficient than replication-based analysis for two-stage genome-wide association studies Nat Genet 2006 14 209 213 10.1038/ng1706 16415888

[B23] Bossini-Castillo L Simeon CP Beretta L Vonk MC Callejas-Rubio JL Espinosa G Carreira P Camps MT Rodriguez-Rodriguez L Rodriguez-Carballeira M Garcia-Hernandez FJ Lopez-Longo FJ Hernandez-Hernandez V Saez-Comet L Egurbide MV Hesselstrand R Nordin A Hoffmann-Vold AM Vanthuyne M Smith V De Langhe E Kreuter A Riemekasten G Witte T Hunzelmann N Voskuyl AE Schuerwegh AJ Lunardi C Airo P Scorza R Confirmation of association of the macrophage migration inhibitory factor gene with systemic sclerosis in a large European population Rheumatology (Oxford) 2011 14 1976 1981 10.1093/rheumatology/ker259 21875883

[B24] Cho JH Gregersen PK Genomics and the multifactorial nature of human autoimmune disease N Engl J Med 2011 14 1612 1623 10.1056/NEJMra1100030 22029983

