# Supplementary material for: KCNA5 gene is not confirmed as a systemic sclerosis-related pulmonary arterial hypertension genetic susceptibility factor
Source: Arthritis Res Ther. 2012 Dec 27;14(6):R273. doi: 10.1186/ar4124 (PMC3674598; doi:10.1186/ar4124)
Supplement: Additional file 1 — Genotype and minor allele frequencies of KCNA5 rs10744676 genetic variant in five European cohorts. This file contains Table S1, showing the genotype and allele distributions of KCNA5 rs10744676 genetic variant in five European cohorts (2,343 SSc cases and 2,690 controls). [file ar4124-S1.DOC]

**Table s1.** Genotype and minor allele frequencies of *KCNA5* rs10744676 genetic variant in 5 European cohorts.

|  |  | **Genotype, N (%)** | | |  | **Allele Test** | |
| --- | --- | --- | --- | --- | --- | --- | --- |
| **Population** | **Subgroup (N)** | **C/C** | **C/T** | **T/T** | **MAF (%)** | ***P*-value*** | **OR [CI 95%]**** |
| Spain | Controls (n=1050) | 12 (1.14) | 196 (18.67) | 842 (80.19) | 10.48 |  |  |
|  | SSc (n=985) | 13 (1.32) | 185 (18.78) | 787 (79.90) | 10.71 | 0.81 | 1.03 [0.84-1.25] |
|  | lcSSc (n=669) | 9 (1.35) | 125 (18.68) | 535 (79.97) | 10.69 | 0.84 | 1.02 [0.82-1.28] |
|  | dcSSc (n=316) | 4 (1.27) | 60 (18.99) | 252 (79.75) | 10.76 | 0.84 | 1.03 [0.77-1.37] |
|  | ACA+ (n=456) | 4 (0.88) | 94 (20.61) | 358 (78.51) | 11.18 | 0.56 | 1.08 [0.84-1.38] |
|  | ATA+ (n=223) | 2 (0.90) | 42 (18.83) | 179 (80.27) | 10.31 | 0.92 | 0.98 [0.70-1.38] |
|  | Fib+ (n=240) | 4 (1.67) | 42 (17.50) | 194 (80.83) | 10.42 | 0.97 | 0.99 [0.72-1.37] |
|  | PAH+ (n=52) | 0 (0.00) | 12 (23.08) | 40 (76.92) | 11.54 | 0.73 | 1.12 [0.60-2.07] |
| The Netherlands | Controls (n=326) | 6 (1.84) | 84 (25.77) | 236 (72.39) | 14.72 |  |  |
|  | SSc (n=174) | 4 (2.30) | 38 (21.84) | 132 (75.86) | 13.22 | 0.52 | 0.88 [0.60-1.29] |
|  | lcSSc (n=130) | 3 (2.31) | 30 (23.08) | 97 (74.62) | 13.85 | 0.73 | 0.93 [0.62-1.41] |
|  | dcSSc (n=44) | 1 (2.27) | 8 (2.27) | 35 (18.18) | 11.36 | 0.40 | 0.74 [0.37-1.49] |
|  | ACA+ (n=43) | 2 (4.65) | 10 (23.26) | 31 (72.09) | 16.28 | 0.70 | 1.13 [0.61-2.08] |
|  | ATA+ (n=46) | 2 (4.35) | 12 (26.09) | 32 (69.57) | 17.39 | 0.50 | 1.22 [0.68-2.18] |
|  | Fib+ (n=81) | 0 (3.70) | 24 (18.52) | 90 (77.78) | 12.96 | 0.57 | 0.86 [0.52-1.43] |
|  | PAH+ (n=24) | 1 (4.17) | 6 (25.00) | 17 (70.83) | 16.67 | 0.71 | 1.16 [0.53-2.55] |
| Italy | Controls (n=360) | 3 (0.83) | 66 (18.33) | 291 (80.83) | 10.00 |  |  |
|  | SSc (n=334) | 4 (1.20) | 64 (19.16) | 266 (79.64) | 10.78 | 0.63 | 1.09 [0.77-1.54] |
|  | lcSSc (n=241) | 3 (1.24) | 42 (17.43) | 196 (81.33) | 9.96 | 0.98 | 1.00 [0.68-1.46] |
|  | dcSSc (n=93) | 1 (1.08) | 22 (23.66) | 70 (75.27) | 12.90 | 0.25 | 1.33 [0.81-2.18] |
|  | ACA+ (n=150) | 3 (2.00) | 29 (19.33) | 118 (78.67) | 11.67 | 0.43 | 1.19 [0.77-1.82] |
|  | ATA+ (n=135) | 0 (0.00) | 24 (17.78) | 111 (82.22) | 8.89 | 0.60 | 0.88 [0.54-1.43] |
|  | Fib+ (n=114) | 0 (0.00) | 24 (21.05) | 90 (78.95) | 10.53 | 0.82 | 1.06 [0.65-1.73] |
|  | PAH+ (n=21) | 0 (0.00) | 4 (19.05) | 17 (80.95) | 9.52 | 0.92 | 0.95 [0.33-2.73] |
| Sweden | Controls (n=249) | 3 (1.20) | 71 (28.51) | 175 (70.28) | 15.46 |  |  |
|  | SSc (n=169) | 5 (2.96) | 29 (17.16) | 135 (79.88) | 11.54 | 0.11 | 0.71 [0.47-1.08] |
|  | lcSSc (n=123) | 3 (2.44) | 21 (17.07) | 99 (80.49) | 10.98 | 0.10 | 0.67 [0.42-1.08] |
|  | dcSSc (n=46) | 2 (4.35) | 8 (17.39) | 36 (78.26) | 13.04 | 0.55 | 0.82 [0.43-1.58] |
|  | ACA+ (n=42) | 2 (4.76) | 9 (21.43) | 31 (73.81) | 15.48 | 1.00 | 1.00 [0.53-1.90] |
|  | ATA+ (n=31) | 2 (6.45) | 6 (19.35) | 23 (74.19) | 16.13 | 0.89 | 1.05 [0.51-2.16] |
|  | Fib+ (n=77) | 2 (2.60) | 16 (20.78) | 59 (76.62) | 12.99 | 0.45 | 0.82 [0.48-1.39] |
|  | PAH+ (n=6) | 0 (0.00) | 1 (16.67) | 5 (83.33) | 8.33 | 0.50 | 0.50 [0.06-3.91] |
| The United Kingdom | Controls (n=705) | 13 (1.84) | 180 (25.53) | 512 (72.62) | 14.61 |  |  |
|  | SSc (n=681) | 17 (2.50) | 155 (22.76) | 509 (74.74) | 13.88 | 0.58 | 0.94 [0.76-1.17] |
|  | lcSSc (n=479) | 13 (2.71) | 109 (22.76) | 357 (74.53) | 14.09 | 0.72 | 0.96 [0.76-1.21] |
|  | dcSSc (n=202) | 4 (1.98) | 46 (1.98) | 152 (22.77) | 13.37 | 0.53 | 0.90 [0.65-1.25] |
|  | ACA+ (n=240) | 7 (2.92) | 55 (22.92) | 178 (74.17) | 14.37 | 0.90 | 0.98 [0.73-1.32] |
|  | ATA+ (n=133) | 2 (1.50) | 33 (24.81) | 98 (73.68) | 13.91 | 0.77 | 0.94 [0.65-1.38] |
|  | Fib+ (n=259) | 5 (1.93) | 57 (22.01) | 197 (76.06) | 12.93 | 0.35 | 0.87 [0.65-1.17] |
|  | PAH+ (n=76) | 1 (1.32) | 14 (18.42) | 61 (80.26) | 10.53 | 0.17 | 0.69 [0.40-1.18] |

Controls are used as reference for all comparisons and *p*-values have been calculated for the allelic model; *Chi-Square p-values calculated for the allelic model. **Odds ratio for the minor allele. MAF: minor allele frequency; 95% CI: 95% confidence interval. SSc: systemic sclerosis; lcSSc: limited cutaneous systemic sclerosis; dcSSc: diffuse cutaneous systemic sclerosis; ACA+: anti-centromere autoantibody positive patients; ATA+: anti-topoisomerase autoantibody positive patients; Fib+: lung fibrosis positive patients (HRCT); PAH+: pulmonary arterial hypertension positive patients (right heart catheterization).
